# Identifying barriers and opportunities to facilitate the uptake of whole genome sequencing in paediatric haematology and oncology practice

**DOI:** 10.1186/s12909-024-06219-y

**Published:** 2024-11-06

**Authors:** Michelle Bishop, Aditi Vedi, Sarah Bowdin, Ruth Armstrong, Jack Bartram, David Bentley, Mark Ross, C. Elizabeth Hook, Brian Hon Yin Chung, Parker Moss, David H. Rowitch, Patrick Tarpey, Sam Behjati, Matthew J. Murray

**Affiliations:** 1 Wellcome Connecting Science, Wellcome Genome Campus, Hinxton, UK; 2 Department of Paediatric Haematology and Oncology, Cambridge University Hospitals NHS Foundation Trust, Cambridge, UK; 3 Department of Paediatrics, University of Cambridge, Cambridge, UK; 4 NHS East Genomic Laboratory Hub, Cambridge University Hospitals NHS Foundation Trust, Cambridge, UK; 5 Department of Medical Genetics, Cambridge University Hospitals NHS Foundation Trust, Cambridge, UK; 6 Department of Paediatric Haematology and Oncology, Great Ormond Street Hospital for Children NHS Trust, London, UK; 7 Illumina, Cambridge Ltd, Cambridge, UK; 8 Department of Pathology, University of Cambridge, Cambridge, UK; 9 Department of Paediatric Histopathology, Cambridge University Hospitals NHS Foundation Trust, Cambridge, UK; 10 Department of Paediatrics and Adolescent Medicine, School of Clinical Medicine, Li Ka Shing, Faculty of Medicine, The University of Hong Kong, Hong Kong, Hong Kong SAR China; 11 Hong Kong Genome Institute, Hong Kong, Hong Kong SAR China; 12 Genomics England, London, UK; 13 Wellcome Sanger Institute, Hinxton, Cambridge, UK

**Keywords:** Accessibility, Barrier, Cancer, Paediatrics, Turnaround time, Whole genome sequencing, WGS

## Abstract

**Background:**

The clinical utility of whole genome sequencing (WGS) in paediatric cancer has been demonstrated in recent years. WGS has been routinely available in the National Health Service (NHS) England for all children with cancer in England since 2021, but its uptake has been variable geographically. To explore the underlying barriers to routine use of WGS in this population across England and more widely in the United Kingdom (UK) and the Republic of Ireland (ROI), a one-day workshop was held in Cambridge, United Kingdom in October 2022.

**Methods:**

Following a series of talks, delegates participated in open, round-table discussions to outline local and broader challenges limiting routine WGS for diagnostic work-up for children with cancer in their Principal Treatment Centres (PTCs) and Genomic Laboratory Hubs (GLHs). Within smaller groups, delegates answered structured questions regarding clinical capability, education and training needs, and workforce competence and requirements. Data was recorded, centrally collated, and analysed following the event using thematic analysis.

**Results:**

Sixty participants attended the workshop with broad representation from the 20 PTCs across the UK and ROI and the seven GLHs in England. All healthcare professionals involved in the WGS pathway were represented, including paediatric oncologists, clinical geneticists, clinical scientists, and histopathologists. The main themes highlighted by the group in ensuring equitable access to WGS identified were: lack of knowledge equity between NHS trusts, with a perception of WGS being for research only; and perception of lack of financial support for the clinical process surrounding WGS, including lack of time to take informed consent from patients. The latter also included limited trained staff available for data interpretation, affecting the turnaround time for reporting. Finally, the need for an integrated digital pathway to order, track, and return data to clinicians was highlighted.

**Conclusion:**

At the workshop, the general motivation for including WGS in the diagnostic work up for children with cancer was high throughout the UK, however a perceived lack of resources and education opportunities limit the widespread use of this commissioned assay. This workshop has led to some recommendations to increase access to WGS in this population in England and more widely in the devolved national of the UK and the ROI.

**Supplementary Information:**

The online version contains supplementary material available at 10.1186/s12909-024-06219-y.

## Introduction

Since early 2021, NHS England (NHSE) has commissioned paired whole genome sequencing (WGS) for all patients < 25 years of age with cancer [1]. Whilst some centres around the world offer genome sequencing to children, these efforts are typically single institution projects or confined to those with high-risk tumours [2–8]. In a model similarly adopted by the Genomic Medicine Sweden Childhood Cancer project [9, 10], the NHSE programme provides nationwide equitable access to WGS, which represents the single most informative assay for cancer to aid patient care [11–13]. However, there is a perception that the uptake of WGS for children with cancer has not being accessed routinely, with notable regional variation.

WGS is a test that is accessible for all clinicians involved in the care of children with malignancies. After parental consent is obtained, samples are tested via a decentralised network of seven Genomic Laboratory Hubs (GLHs) that serve the 15 paediatric haematology and oncology units (Principal Treatment Centres; PTCs) in the NHS in England. Successful implementation, however, depends on the requisite infrastructure and multiple health care professionals, including paediatric haematologists and oncologists, pathologists, clinical geneticists, clinical scientists, and technical laboratory staff, comprising genetic technologists and biomedical scientists. The reasons for incomplete uptake of WGS for childhood cancer are not fully understood, but likely relate to the complexities around aligning infrastructure needs, capacity building, and clinical workflows across multiple hospital sites.

To obtain a more systematic understanding of barriers to offering WGS for children with cancer, and to identify potential solutions, an in-person workshop was convened that brought together key stakeholders from across the United Kingdom (UK; England, Wales, Scotland, Northern Ireland), and the Republic of Ireland (ROI), as well as some international participants. The event was hosted by Wellcome Connecting Science in collaboration with the University of Cambridge Department of Paediatrics, Cambridge University Hospital NHS Foundation Trust, East-GLH, Genomics England, and NHSE. Prior to the workshop, a pre-meeting survey was conducted amongst all delegates that confirmed notable regional variation in uptake of WGS for children with cancer within the GLHs which comprise NHSE, in the devolved nations of Scotland, Wales, and Northern Ireland, and ROI. A key component of the event itself was a structured workshop, used to elucidate current and anticipated challenges and opportunities from end users. Here, we provide a qualitative themed analysis of the main barriers and possible solutions identified by participants, to help ensure that every child with cancer in the UK and ROI may benefit from WGS.

## Methods

### Workshop overview

This purpose of the one-day workshop, held on 20 October 2022, was to bring together key stakeholders from all four countries of the UK, and ROI. The day was structured with a morning session, comprised of a series of didactic lectures, and the afternoon session where participants were allocated to small groups to work through a structured questionnaire. The groups then convened for a round-table discussion before the day closed. To allow for free communication, the afternoon workshop followed ‘Chatham House Rules’ [14], namely that neither the identity nor the affiliation of the speakers/participants may be revealed in the dissemination of the findings. The running of this workshop, and collection of data, was approved by the Research Governance Committee (which oversees the Research Ethics Committee) at the Wellcome Sanger Institute, UK.

### Attendees

Using purposive sampling principles, the attendee list was assembled to ensure representation from the PTCs as well as the seven GLHs, coverage of all healthcare professionals involved in the clinical pathway, and input from across the UK and ROI, as well as centres of excellence internationally. Personal invitations were sent by PT, SB, and MJM via email. Invitees who were unable to attend due to scheduling conflicts were asked to identify a suitable alternative. An a priori decision was made to cap the number of attendees to 60. This balanced the need for sufficient representation from key organisations with the practicalities of capturing feedback from a diverse audience.

### Data collection

In the afternoon session, attendees in breakout groups of seven or eight worked through a series of structured questions (see Supplementary Table 1). These questions were informed by the COM-B model of behaviour (see Table 1) [15], where capability (C), opportunity (O), and motivation (M) are perceived as the three key factors capable of changing behaviour (B). The COM-B model is widely used to identify factors that need to be considered for any behaviour change intervention to be effective [16–18], such as the implementation of WGS into clinical care. The questions that explored capability were focussed on identifying where education and training could facilitate uptake of WGS. That is, they were to identify education and training needs and not intended to be a subjective assessment of workforce competence. Responses to the structured questions were captured by a scribe in each group. During the round-table discussion, field notes were taken by MB to capture key discussion points and verified via an audiotape of the session. The findings presented were de-identified, removing any information around an individual’s clinical profession or place of work, to align with Chatham House Rules.

**Table 1:** COM-B model of behaviour

Factors	Targets to influence behaviour change
**Capability (C)**	Targets may include understanding why and how to make the change, having the competencies needed to implement and sustain the change.
**Opportunity (O)**	Targets may include having the financial and material resources to make the change, having sufficient time to enact the change; exposure to social or other prompts to undertake the behaviour; having a supportive work culture to encourage the behaviour change.
**Motivational** (M)	Targets may include truly wanting or needing to engage in the behaviour, having habits and routines, and values and identity that embrace the behaviour.

### Data analysis

The written responses from the groups and the notes from the group discussion were typed and uploaded to NVivo 12, the qualitative data analysis computer software package. The text was analysed through deductive content analysis [19] initially by MB and reviewed by MJM, AV, and PT. The predefined categories were informed by the COM-B model and coded as facilitators or barriers.

## Results

Sixty people attended the workshop. Of those that attended, the largest single group (*n* = 26) were paediatric oncologists, including those that had an academic appointment, making up 43% of attendees. The remaining 34 (57%) attendees included clinical scientists and bioinformaticians (*n* = 11), paediatric pathologists (*n* = 8), paediatric haematologists (*n* = 5) and clinical geneticists (*n* = 5). The other five individuals were from key organisations and/or speakers. While all countries in the UK and ROI were represented, the vast majority (83%) were from England. The morning session included international representation from Hong Kong to provide a case study of successful implementation of WGS in a non-NHS setting.

### Key education and training targets to support uptake of WGS

While all attendees recognised the work of NHSE in providing equity of access to WGS, the group felt the same could not be said about the knowledge base of the workforce. This was felt even at the level of understanding the clinical utility of WGS. Whilst the group recognised many oncologists and haematologists are “*very invested in WGS”* this was not universal, with others *“not even aware of the existence of WGS”.* Attendees acknowledged the disparity in the availability of local expertise. The phrase *“lack of knowledge equality”* was used to describe the difference in knowledge (and skills) between NHS Trusts, departments and even colleagues within the same department. All attendees believed this inequality would, inevitably, impact the equity of access to WGS.

Several education and training needs were identified by the group. This included process knowledge as well as clinical skills. Full details are outlined in Table 2.

**Table 2:** Education and training needs

Topic	Details	Target audience
Process knowledge	Understanding the processes for requesting WGS including how to use the National Genomics Information System (NGIS) and to complete the Record of Discussion (RoD), the latter being the WGS-specific consent form.	Healthcare professionals involved in facilitating WGS.
Consent conversation	Key information to include in the consent conversation, including the potential to identify germline (inherited) variants.	Healthcare professionals who will undertake the consent conversation.
Variant interpretation	Calling biomarkers for clinical trials	Clinical Scientists
	General principles and terminology to participate effectively in Genomic Tumour Advisory Boards (GTABs).	Pathologists, haematologists, oncologists
Return of results	How to respond to the most common questions from parents, including the ‘why me’. How to return germline variant results.	Healthcare professionals who are returning WGS tumour results.

A small group of attendees (representing approximately 20% of the audience) challenged the *status quo* on the current model of workforce development in genomics. There was a suggestion that some clinical tasks, such as taking and recording consent for genomic tests, should require mandatory (or, as stated “*formal*”) training and assessment of competence, which is in place in some NHS Trusts, but is not routine practice.

Whilst all attendees agreed that workforce development activities were needed, and wanted by healthcare professionals, about half acknowledged that clinicians may already possess relevant knowledge and skills but lack confidence and clinical experience. This group felt that any education and training activities should also provide clinicians with protected time in which to practise their skills by, for example, through role-play.

Throughout the discussion, it became clear to both workshop attendees, and facilitators, that access to education and training differed depending on where healthcare professionals worked, with some GLH/Genomic Medicine Service Alliances (GMSA) and Trusts providing more workforce development opportunities around consent and variant interpretation (amongst other topics) than others (*“we have sessions on this*,* don’t you?”)*.

### Other barriers impeding implementation and/or uptake of WGS

The commissioning of WGS for paediatric malignancies by NHSE was lauded by all attending the workshop. However, for those working in Scotland, Wales, Northern Ireland, and the ROI, funding this service was seen as the priority before anything else could, or should, be considered.

For those working in England, the perpetuating theme from both the group work and subsequent discussion was that opportunities to implement WGS at a Trust and/or pathway level have, from the clinician’s perspective, not been built into existing systems/infrastructures. This includes a lack of systematic clinical pathways and sufficient resources - both financial and people. When the question around resources was put to the groups, the consensus view was there was just simply not enough, with the clear message from one group being *“No*,* a resounding no”*. The perception is that most of the current activity is happening due to the ‘goodwill’ of a few people. As summed up by one of the groups, the view from those involved with implementing this test is that: *“The test is funded but the clinical process is not.”* Specific examples included staff capacity across the pathway (*“not enough time for interpretation*,* not enough scientists full stop”)* and properly resourcing Genomic Tumour Advisory Boards (GTABs) *“we have established specific GTABs*,* but clinician time to attend GTABs (is) not funded”)*.

Not having sufficient time was a recurring theme, with a particular emphasis on the consent process. This was both in how long it took to obtain consent (or was perceived to take) and which, if any, healthcare professionals then have the time to obtain and record consent. If these issues were not addressed, all participants felt this would create an insurmountable barrier to ubiquitous uptake of WGS across the NHS.

The oncologists and haematologists were perplexed as to why the consent process for WGS needs to be separate to standard diagnostic tests and, as such, take additional time to complete. For many of these clinicians, their patients are unwell and simultaneously having other tests, procedures, and/or being enrolled for clinical trials - all of which require verbal and/or formal written consent. This means that clinicians and parents are faced with multiple consent processes and forms at a very stressful time for parents/guardians. It was felt that parents/guardians would benefit from simplification of the consent process, or for consent to occur in a staged manner. For example, one group commented it would be easier for the parents to provide generic consent for collection of the germline sample and then have the consent conversation for analysis after treatment has commenced. As they stated, *“you can have a much more sensible conversation four weeks into treatment. This way you prioritise consent for tests that will impact first line treatment*,* rather than do everything at once”.* For other attendees, specifically those from a genetic/genomic professional background, the recognition that WGS is the only tumour assay where germline variants are routinely explored justified having separate consent processes, although recognition of overburdening parents at such a stressful time was appreciated.

Despite some tension regarding single or multiple consent processes, attendees all agreed that obtaining consent for WGS significantly impacted clinicians’ ability to deliver other aspects of patient care at the critical diagnostic timepoint. A suggested solution was to employ dedicated staff for this role. However, there was caution in how this should be implemented. As stated by one attendee, *“There needs to be investment in staffing*,* not just taking resources from another part of the service.”*

Time was also seen as a barrier for the interpretation of variants identified by WGS, which had a knock-on effect on the turnaround time (TAT) for reporting. It was recognised that this is most likely a capacity issue within the laboratories. However, some of the clinical scientists in the room reported an additional physical barrier that would also impact TAT; that of not being able to access key academic journals through their institution *(“It just takes more time*,* time to find a colleague who has an academic appointment who has the time to resource the article for you. It just all adds to the delay in returning results”)*. This delay in access to critical data/resources that may influence variant interpretation will inevitably have an impact on TAT.

There was agreement from all non-clinical genetics attendees that lack of access to specialist genetic clinical services was also a major factor in the ability to universally implement WGS. This ranged from the limited ability of clinical geneticists to provide input into GTABs, to the very long waiting lists for clinical appointments after the detection of a germline variant *(“the waiting list can be up to a year”).* For some oncologists and haematologists at the workshop, this meant that they feared receiving a result which reported a germline variant, as they knew how long it could be until the parents/guardians were able to access specialist clinical genetics support (“*should we be offering this test if we can’t get appropriate clinical support to deal with the results?”)*.

The final area that was discussed were processes and systems that would not only operationalise the end-to-end pathway, but also facilitate implementation of WGS. This included: developing user-friendly digital systems that supported the ordering of tests and tracked samples as well as having a record of consent (*“need better information technology to flag missing consent*,* samples”);* defined clinical pathways *(“Have proper process maps and clinical pathways so we all know who is responsible for the task”)* and national guidance for clinical trials; and inter- and intra-Trust tissue transport logistics that ensured samples arrived at the relevant laboratory in a safe and timely manner (“*in some cases consultants are transporting samples between hospitals”*).

There was a strong sense from all of those at the workshop that it will be much easier to onboard clinicians to offer and consent for WGS when all the pathways, systems, and guidelines are embedded in hospital Trusts.

### Motivation to offer WGS

As a group, the attendees at the workshop considered introducing WGS as a positive step and that the implementation of the test will achieve desired outcomes. However, there was a recognition by the oncologists and haematologists in the room that some of their colleagues do not have the same vision. Several theories were suggested as to why this may be this case. These included:

- A general lack of awareness that WGS is an option. Attendees suggested WGS needs to be included in relevant clinical checklists to remind people of the availability of the test and therefore ensure it is considered *(“Suggest including in a checklist or making this part of tumour board discussions for each new patient. Could be included in ward rounds*,* pre-clinic meetings*,* pre-ward round meetings”)*.
- Their colleagues’ current level of trust and ‘comfort’ with standard-of-care (SOC) testing – and questioning the benefits of WGS over SOC. This was seen as an issue that needed to be addressed as the tumour samples for this patient cohort are often small. Therefore, it was felt that there needs to be a push to prioritise WGS for testing rather than an optional extra if there was sufficient tissue to test.
- Finite resources within the NHS. There may be a tension between taking resources from other services to implement WGS, when SOC testing is already being undertaken.
- A perceived lack of support from Trust management (in some Trusts) which may influence the distribution of resources and provide a subliminal message on the importance of WGS *(“Our Trust has not bought into NGS (Next Generation Sequencing)*,* they have different priorities)*.

Some of the oncologists felt that there needs to be a shift in mindset as WGS is still seen by many in the NHS *“as research & discovery rather than clinical test”*. As such, WGS needs to be ‘marketed’ to clinicians as a routine SOC clinical test that is available now.

There was also universal recognition that people will be influenced by the views of others within the GTAB, whether they be positive or negative, towards the use of WGS. Therefore, it was felt that there is a need for a widespread campaign illustrating what WGS can offer compared with SOC testing. This could include, for example, the sharing of good practice through knowledge exchange activities like, for example, virtual ‘Grand Rounds’ or national GTABs.

## Discussion

**Fig. 1:**
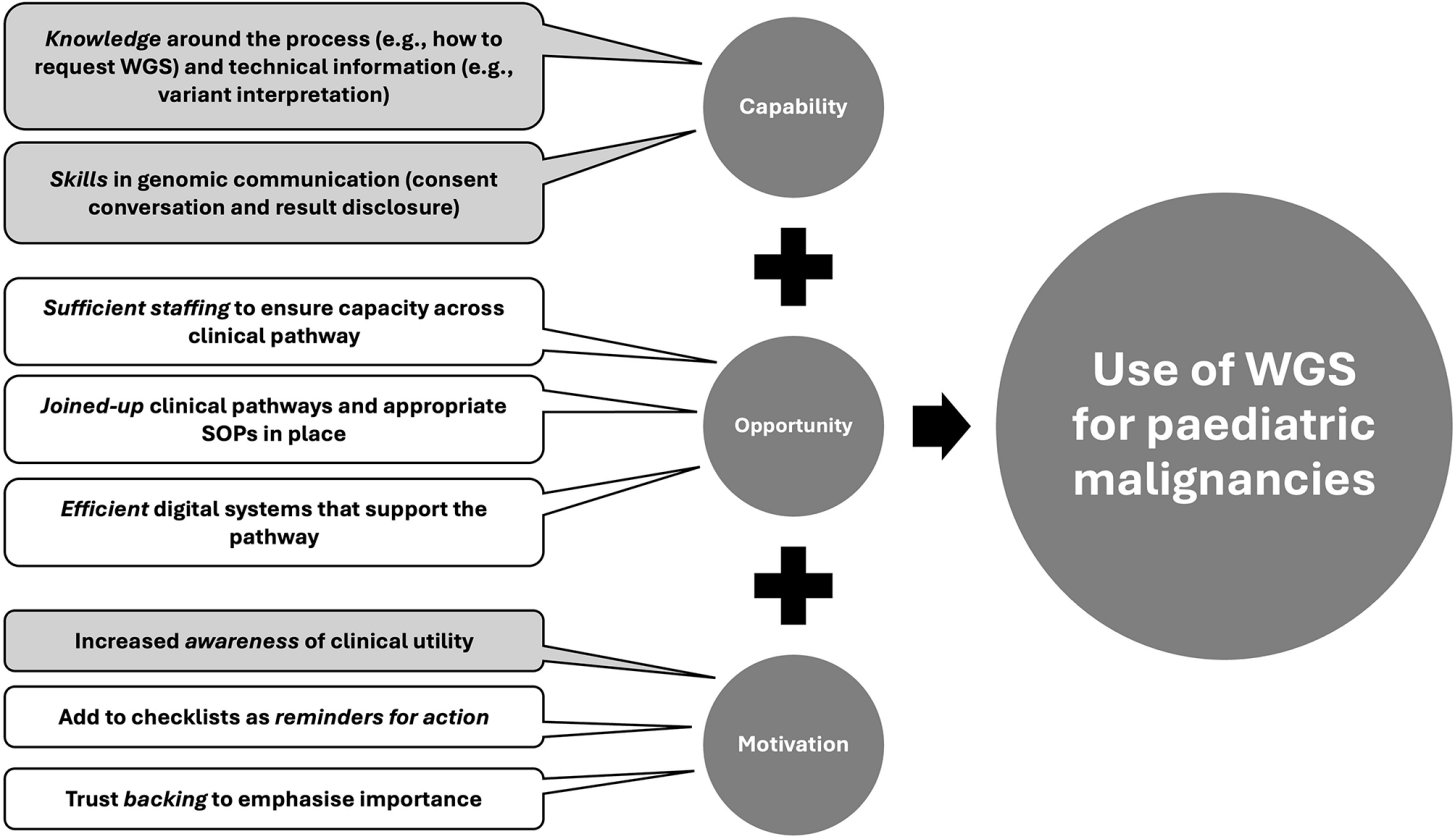
Overview of paediatric whole genome sequencing (WGS) workshop findings. The ‘capability’, ‘opportunity’ and ‘motivation’ factors that workshop participants identified need to be in place to increase the use of WGS for paediatric malignancies. The factors that can be addressed through education and training are shaded. The remainder require input at a Trust and/or central NHS level. Key: SOP = standard operating procedure

The aim of this workshop was to identify the current and anticipated challenges and opportunities to facilitate the uptake of WGS for children with malignancies. Through this process, we have been able to categorise the findings as those where education and training can be used to overcome identified barriers, and where further consideration may need to be made around the operationalising this clinical pathway (see Fig. 1).

### Where education and training can make a difference

Despite WGS being routinely available in this context in the NHS in England for over 18 months at the time of the workshop, our findings suggest there is still a requirement for raising awareness amongst clinicians. Surprisingly, the view of attendees was that their colleagues do not view WGS as SOC, even though it is commissioned and resident in the cancer test directory [20]. There is a sense that many clinicians still see WGS as belonging solely in the domain of research projects and not as a test routinely in the clinical setting. If this is truly the view of even a minority of NHS clinicians, this misconception about the availability of WGS needs to be addressed so that clinicians appreciate that this test that is available within the NHS as SOC for patients < 25 years with cancer and can deliver clinically insightful variants inaccessible via alternate assays.

Attendees also felt that there is a clear ‘lack of knowledge equity’ across England and the UK more widely. Knowledge deficits need to be addressed to ensure all relevant healthcare professionals’ base-line knowledge is sufficient to ensure patients receive the same access to diagnostic tests regardless of geographical location. Alongside this, it is believed that many clinicians lack confidence and clinical experience, and traditional education and training events may not meet these training needs. Education and training providers need to provide a way for clinicians to ‘practice’ in a safe environment where they can make mistakes - such as using role-play or standardised patients - either in person or in a virtual learning environment [21–23].

While not explicitly stated by workshop attendees, it is recognised that a lack of data on the clinical utility of WGS versus SOC may also be a barrier to uptake. WGS analysis is evolving, and the algorithms and interfaces which provide the results are under active and ongoing development. These enhancements will expedite and improve the scientific analysis and clinical interpretation of what is perceived as a complex assay, and disseminating this information to the clinical workforce can only expedite uptake.

The suggestion of a national standardised programme of mandatory training and assessment of competence would provide a structured way to address these training needs. This will also provide clinicians (and patients) with the assurance that any healthcare professional providing this service, regardless of professional background or geographical location, is equipped with the relevant knowledge and skills. To deliver this programme of training, there will need to be a corresponding equity of access to education and training across the board. Alongside work that is being coordinated at a national level in England to facilitate equity of access to education and training resources through the work of NHS England Genomics Education [24], GLHs/GMSAs and Trusts in the devolved nations will need to work together and share resources to ensure no clinician is disadvantaged in accessing appropriate training due to their location. Leveraging existing education and training frameworks, such as those for paediatric oncologists, will effectively disseminate crucial information about WGS.

A centralised resource, such as a national standardised GTAB, may be a consideration for analysis, interpretation of results, and clinical recommendations to increase access to WGS as an important clinical and research tool. Indeed, such national tumour boards have been suggested for different paediatric cancers to provide treatment recommendations. However, these boards would carry substantial resource implications, including the need for appropriate financing to support time and infrastructure requirements. Based on clinician dedication and goodwill, in the UK there are existing Children’s Cancer and Leukaemia Group (CCLG) National Advisory Panels (NAPS) which provide treatment suggestions, but which are not formally funded. Thus, at present, without more dedicated funding, the centralised sequencing approach with devolved analysis/interpretation is the most pragmatic model, alongside the development of education workshops/events.

### Other areas that need to be addressed to improve uptake

For the other UK nations (outside of England) and ROI it was clear that the first barrier that must be addressed is sustainable funding for the service. For those working in England, attendees identified their perception that investing in the test will not correlate with adoption into clinical practice unless investment is also made for the clinical process. Otherwise, it was felt that the service relies on ‘goodwill’ which is not sustainable and will not translate into an equitable service. Our findings indicate attendees wanted resource investment across the end-to-end pathway including capacity to take consent and through to increased access to clinical genetics services.

While the conversations on consent during the workshop focused on consent for the clinical test, WGS also provides an opportunity for patients/parents/guardians in England to consent for their data and samples to be included within the National Genomic Research Library (NGRL), which is overseen by Genomics England. Consenting to the NGRL is not mandatory, and indeed around 3% of cancer patients who receive WGS via the NHS Genomic Medicine Service decline to sign the research consent forms (personal communication, Parker Moss). The rationale for obtaining ‘research consent’ is that beyond the clinical purpose of the test, consented patient genomic and clinical data are de-identified and stored in the NGRL. Through linking national health datasets, this research resource follows the patient’s clinical results for the rest of their lives, enabling the study of longitudinal outcomes by genotype and by intervention. This creates material research upside to the test beyond its clinical benefit, but this upside does require the patients to be informed and to consent to the storage and follow-up of their data, further complicating the research conversation.

### Next steps

The workshop findings identified several actions that can be taken to improve uptake of WGS. Firstly, the topics outlined in Table 2 provide a broad curriculum for education and training that providers developing and delivering sessions around WGS for the different health professional groups can use to ensure coverage of relevant knowledge and skills within any education sessions. Although the authors’ goal was a coordinated education program within 12 months of the workshop, successful implementation depends on collaboration with partners with differing priorities. Nonetheless, significant strides have been made; SB, MJM, PT, and AV delivered a teaching session to the CCLG Paediatric Oncology Trainee Group (POTG) in May 2023 as part of their scheduled learning programme, using case studies to cover the topics in Table 2. Efforts will continue with key stakeholders including NHS England Genomics Education and the Royal Colleges who are charged with delivering training curriculum for paediatric oncologists and haematologists to ensure the relevant health care professionals receive timeline and pertinent education and training in this topic.

Regarding the other barriers identified, there were many providers within the room that are achieving excellence in certain parts of the clinical pathway, even if no institutions appear to have a perfect end-to-end process. For example, through the workshop, participants shared examples of best practice and potential ways of working, such as using centralised model for analysis an interpretation of results as well as and regional tumour boards to support implementation. This sharing of this type of information was seen by many as an unexpected benefit of attending the workshop, and as such participants requested annual or bi-annual meetings. Bringing this groups of attendees together on a regular basis would provide an avenue for collective problem solving, essentially establishing a UK and ROI community of practice in this area [25].

The effectiveness of these interventions will be evaluated based on the volume of WGS tests ordered and the equitable access provided across the UK. Data from NHS England, along with counterparts in Scotland, Wales, and Northern Ireland, as well as the ROI, will track test uptake, offering a valuable objective measure to assess behavioural changes over time.

## Conclusion

This workshop provided an opportunity and forum for change leaders in the UK and ROI to have a focused discussion on the implementation of WGS in paediatric haematology and oncology practice. Using structured questions within a group discussion ensured that all areas of the clinical pathway and implementation process were covered, and exposed many perceived barriers which were not commonly known across the whole group. Outcomes from this workshop have identified where education and training can improve uptake. However, for education and training to have an impact on behavioural change, consideration also needs to be made for appropriate operational changes. This will facilitate the most widespread and equitable uptake of WGS in this cohort, with the aim of allowing real-time changes in clinical management and ultimately improving patient outcomes.

## Electronic supplementary material

Below is the link to the electronic supplementary material.

Supplementary Material 1

## Data Availability

No datasets were generated or analysed during the current study.

## Abbreviations

CCLG: Children’s Cancer and Leukaemia Group
GLH: Genomic Laboratory Hubs
GMSA: Genomic Medicine Service Alliance
GTAB: Genomic Tumour Advisory Board
NGRL: National Genomic Research Library
NHS: National Health Service
NHSE: National Health Service England
POTG: Paediatric Oncology Trainee Group
PTC: Principal Treatment Centre
ROI: Republic of Ireland
SOC: Standard of Care
TAT: Turnaround Time
UK: United Kingdom
WGS: Whole Genome Sequencing
