# Supplementary material for: Identifying barriers and opportunities to facilitate the uptake of whole genome sequencing in paediatric haematology and oncology practice
Source: BMC Med Educ. 2024 Nov 6;24:1273. doi: 10.1186/s12909-024-06219-y (PMC11542304; doi:10.1186/s12909-024-06219-y)
Supplement: Supplementary file 1 — Supplementary Material 1 [file 12909_2024_6219_MOESM1_ESM.docx]

**Supplementary Table 1: Questions based on COM-B behavioural change model.**

The COM-B behavioural change model considers different factors which influence achieving a desired change in behaviour – in this case offering whole genome sequencing. The factors can be categorised as either ‘capability’ (C), ‘opportunity’ (O)’ or ‘motivation’ (M).

**Capability** targets include understanding why and how to make the change, and having the competencies needed to implement and sustain the change.

**Opportunity** targets include having the financial and material resources, having sufficient time; exposure to social or other prompts; and having a supportive work culture.

**Motivational** targets include truly wanting or needing to engage in the behaviour, having habits and routines, and values and identity that embrace the behaviour.

In the first part of the afternoon workshop, attendees answered specific questions listed below, to identify which aspects of doing the behaviour (i.e., approaching, and consenting patients and their families for WGS) could potentially be targeted through education and training, to achieve this desired behaviour. For each question, attendees were asked to choose a ‘yes’, ‘to a limited degree’, or ‘no’ response and then provide a descriptive response.

| **Questions: Please consider this from your perspective, your colleagues, and your hospital and/or Trust. We have used the term ‘your colleagues’ or ‘they’ as a shorthand throughout the questionnaire.** | |
| --- | --- |
| **Capability: psychological** | |
|  | Do your colleagues fully understand why it (WGS) is important – do they understand the benefits? |
|  | Do your colleagues know how to?  o Identify patients suitable for WGS  o Discuss of test and possible results (information sheets)  o Obtain consent  o Confirm if fresh frozen tissue available  o Obtain germline sample (blood or skin biopsy)  o Process the sample  o Interpret the results  o Feedback the results  o Provide or refer for ongoing clinical management |
|  | Are your colleagues likely to remember to do the behaviours above? |
| **Opportunity: physical** | |
|  | Do your colleagues have the time to do the behaviour? |
|  | Do your colleagues have the financial resources to do the behaviour? |
|  | Do they have the material support required (e.g., equipment, staff) |
|  | Are there procedures or ways of working that encourage the behaviour? |
|  | Are there triggers to prompt them, e.g., have reminders at strategic times? |
| **Opportunity: Social** | |
|  | Are social influencers likely to facilitate (positive) or hinder (negative) behaviour (e.g., peers, managers, patients etc) – please specify which one. |
|  | Are there people around in their department doing it? |
| **Motivation: reflection** | |
|  | Does doing the behaviour conflict with other behaviours? |
|  | Do your colleagues believe that it would be a good thing to do? (e.g., do they have a strong sense that they should do it?) |
|  | Are there incentives to do the behaviour? |
|  | Is this behaviour seen as normal and commonplace? |
|  | Do they have effective plans for doing the behaviour? |
|  | Are your colleagues confident that performing the behaviour will achieve the desired benefits/outcome? |
| **Motivation: automatic** | |
|  | Can your colleagues be led to develop a habit of doing the behaviour (e.g., have a pattern of doing it without thinking about it) |
